# Simulation of the Effect of Material Properties on Soft Contact Lens On-Eye Power

**DOI:** 10.3390/bioengineering6040094

**Published:** 2019-10-09

**Authors:** Joshua Moore, Bernardo T. Lopes, Ashkan Eliasy, Brendan Geraghty, Richard Wu, Lynn White, Ahmed Elsheikh, Ahmed Abass

**Affiliations:** 1 School of Engineering, University of Liverpool, Liverpool L69 3GH, UK; sgwmoore@liverpool.ac.uk (J.M.); blopesmed@gmail.com (B.T.L.); eliasy@liverpool.ac.uk (A.E.); bren@liverpool.ac.uk (B.G.); elsheikh@liverpool.ac.uk (A.E.); 2 Department of Ophthalmology, Federal University of Sao Paulo, Sao Paulo 04021-001, Brazil; 3 Department of Optometry, Central Taiwan University of Science and Technology, Taichung 40601, Taiwan; richard.wu@brightenoptix.com; 4 College of Optometry, Pacific University, Forest Grove, OR 97116, USA; 5 UltraVision CLPL, Leighton Buzzard, Bedfordshire LU7 4RW, UK; Lynn@lwvision.co.uk; 6 School of Biological Science and Biomedical Engineering, Beihang University, Beijing 100191, China; 7 National Institute for Health Research (NIHR) Biomedical Research Centre at Moorfields Eye Hospital, NHS Foundation Trust and UCL Institute of Ophthalmology, London EC1V 2PD, UK

**Keywords:** contact lenses, hydrogel, silicone hydrogel, optical power, on-eye, off-eye

## Abstract

Purpose: To evaluate the variation in the optical power achieved following soft contact lens eye fitting for spherical and cylindrical lenses with differing hydrogel material properties. Methods: Uniaxial tensile tests were performed on four hydrogel materials 77% water-content (w-c) hydrogel, 74% w-c blue silicone hydrogel, 74% w-c clear silicone hydrogel, and 64% w-c clear hydrogel (shortly referred to as H77p0, SiH74p5-blue, SiH74p5-clear, and H64p0-clear), under loading conditions that would be expected in vivo. Finite element models of the cornea and contact lens interaction were generated using spherical and cylindrical lenses with powers varying from −10 to +20 D; overall diameters of either 13.5, 14.0, or 14.5 mm; and with material properties matching those determined through experimental testing. Results: The moduli of elasticity for each of the tested hydrogel materials were 0.195 ± 0.027 MPa, 0.277 ± 0.019 MPa, 0.279 ± 0.01 MPa, and 0.457 ± 0.013 MPa for H77p0, SiH74p5-blue, SiH74p5-clear, and H64p0 respectively. The calculated values of effective power change (EPC) showed strong negative correlations with lens power. This was particularly apparent in the higher end of the lens power spectrum (over +5 D), where each of the materials demonstrated a highly linear reduction in EPC with increased lens power. Conclusions: Soft contact lenses composed of a stiffer hydrogel are far more resilient to changes in EPC across the lower end of the lens power spectrum (−10 to +5 D). Beyond this range, the material choice does not have a significant effect on the EPC.

## 1. Introduction

Consistent development and evolution of new contact lens materials have been observed throughout the last century [1,2]. Considering the mechanical properties of these new materials is vital for sufficient functionality of the soft contact lenses [3]. If this consideration is not afforded, the lenses may fail through fracture or other mechanisms, leading to an overall loss in optical performance [4,5]. Unpredictable changes in optical power, as a result of poor design, can be observed during the fitting process [6].

Although the diameter and curvature of prescribed contact lenses can be tailored to suit a patient’s individual refractive needs, it is not yet possible to account for the many interactions that affect the surface of the contact lens throughout the fitting procedure. During this process, the soft contact lens will conform to the shape of the cornea, thus changing its overall refractive power [7,8]. This change in lens geometry is further influenced by both the movement of the eyelid and the surface tension created by the tear film [9,10]. The combination of these factors leads to a change in contact lens geometry and subsequent change in the refractive power that is highly anisotropic and often difficult to predict [11]. It has been previously hypothesised by Janoff and Dabezies [12] that the flexure of the soft contact lens is synonymous with the bending of a beam. This theory implies that, as is true in Euler–Bernoulli beam theory [13], the material properties of the contact lens directly affect the magnitude of flexure and the subsequent change in its refractive power.

The study presented in this paper utilised a combination of finite element and light ray tracing analyses to determine the change in refractive power in contact lenses composed of various hydrogel materials with a differing modulus of elasticity.

## 2. Materials and Methods

### 2.1. Participants

In this record review study, fully anonymised secondary data were user and according to the University of Liverpool’s Policy on Research Ethics, ethical approval was unnecessary. Nevertheless, the study followed the tenets of the Helsinki Declaration of 1964 which was revised in 2013. In this study, clinical data of one left eye of a 24-year-old male patient with an average eye-lens was used (Flat Sim-K = 43.8 D) [14]. The selection of suitable eyes for inclusion in the study was carried out following the eye topography population study of Gilani [15]. The detailed procedure of taking measurements was described in our previous openly accessed publication [16].

### 2.2. Uniaxial Tensile Testing

Uniaxial tensile tests were performed on four different non-ionic hydrogel materials namely, 77% water-content (w-c) hydrogel, 74% w-c blue silicone hydrogel, 74% w-c clear silicone hydrogel, and 64% w-c clear hydrogel (shortly referred to as H77p0, SiH74p5-blue, SiH74p5-clear, and H64p0-clear), Table 1.

The testing procedure involved taking three samples of each material, measuring their corresponding stress–strain behaviour and using these values to compute their average modulus of elasticity. The tests were conducted in the Biomedical Engineering Lab at the University of Liverpool (Liverpool, UK) using an Instron 3366 material testing machine with BlueHill 3 control software (Instron, Massachusetts, USA).

**Figure 1:**
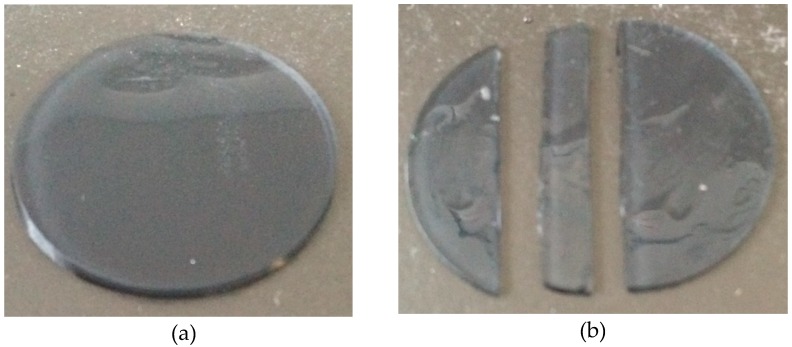
Image of an H77p0-clear hydrogel sample (77% water-content (w-c) hydrogel) before (**a**) and after (**b**) being cut into a strip.

Samples of each material were prepared at UltraVision CLPL (Leighton Buzzard, Bedfordshire, UK) by clipping dehydrated hydrogel blanks into a plastic contact lens blank holder. A flat-mount brass chuck was heated to 60 °C and the mounting end dipped into melted blocking wax (Nexgen Optical, Oxon, UK). The hydrogel blank was attached to the brass chuck using a static blocking tool (Larsen Equipment Design, Seattle, WA, USA). The blocking tool attached the hydrogel blank to the brass chuck by applying a constant force between the two until the blocking wax cooled. The plastic blank holder was then removed by hand, and the brass chuck, with hydrogel attached, was mounted on an Optoform 40 lathe (Sterling Ultra Precision, Wokingham, UK). The computer numerically controlled (CNC) lathe was programmed to reduce the blank thickness to 0.55 mm. Once the desired thickness had been achieved, the brass chuck with hydrogel attached was placed into an ultrasonic paraffin bath. This allowed the hydrogel to detach from the mount, whilst also removing any excess wax. The hydrogel discs were stored for eight hours in 0.90% borate-buffered saline solution (Sigma Aldrich, Missouri, USA) to hydrate and swell the material. A double-bladed cutting tool was used to cut the samples along their diameters, to achieve the strips necessary for the tensile test, Figure 1. The dimensions of each sample (length, width, and thickness) were determined using a digital Vernier calliper (D00352, Duratool, Taiwan). These dimensions were measured at three different locations along the sample length and then averaged.

**Figure 2:**
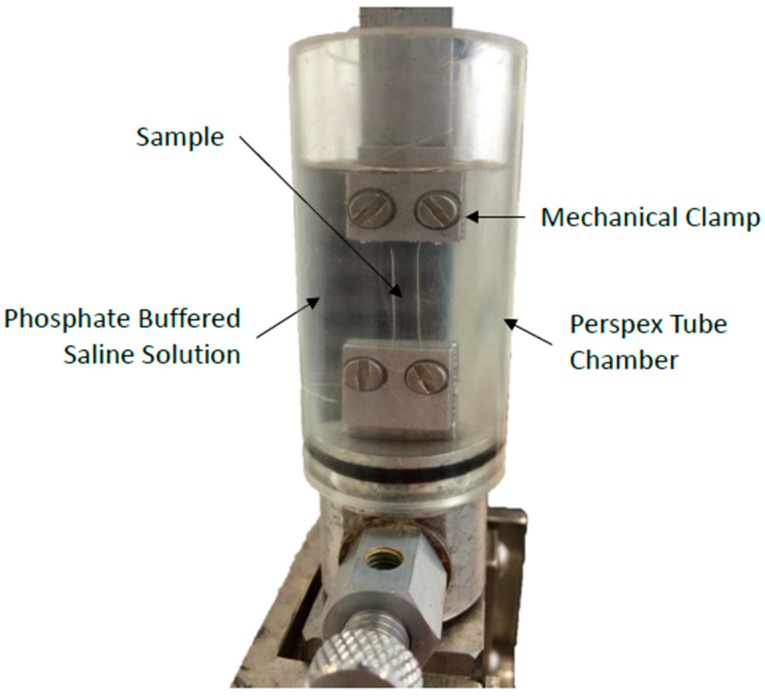
Setup used for the tensile testing of the hydrogel materials.

The samples were secured in a set of mechanical clamps designed for use with the tensile testing machine. In order to maintain hydration throughout the testing procedure, the samples were submerged in a Perspex chamber filled with phosphate-buffered saline solution (Sigma Aldrich, St. Louis, MO, USA), Figure 2. The samples were strained at a rate of 10% min^−1^ until failure, and the values of the force, *F*, at specified time increments were recorded and converted into tensile stress, $\sigma_{t},$ values by dividing them by the sample’s initial cross-section area, $A_{0}$ (Equation (1)) [17].

(1) $$\sigma_{t}=\frac{F}{A_{0}},$$

In the same time increments, the change in sample length, $\Delta L=L_{1}-L_{0},$ was recorded by measuring the instantaneous length, $L_{1},$ and dividing ΔL by the initial length of the strip, $L_{0},$ in order to calculate the strain, ε (Equation (2)).

(2) $$\varepsilon =\frac{\Delta L}{L_{0}},$$

The calculated values of stress were plotted against strain, and the modulus of elasticity was calculated by computing the gradient of the graph (Equation (3)).

(3) $$E=\;\frac{\Delta \sigma_{t}}{\Delta \varepsilon },$$

### 2.3. Finite Element Modelling

**Figure 3:**
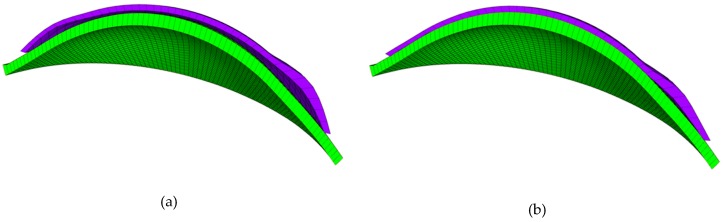
Finite element model of the contact lens (purple) and cornea (green). This model is shown before (**a**) and after (**b**) fitting.

Finite element models of the cornea–lens system were generated using a custom-built MATLAB program. The finite element model consisted of two components: the anterior eye and the contact lens which were connected by a single interface, Figure 3. Two mathematical domains, $\Omega_{1},{\;\Omega }_{2},$ were defined for each of the components respectively. The surfaces of these domains were denoted $\partial \Omega_{1},\partial \Omega_{2}$. The interface upon which the two surfaces were in contact was denoted $\Gamma_{c}$, where $\Gamma_{c}$ is simply the set intersection between the two surfaces. It was assumed that the two surfaces were impenetrable and that the coefficient of friction was a constant value of 0.01 across the entirety of $\Gamma_{c}$ [18]. The traction acting on the interface, $\Gamma_{c},$ of the surfaces $\partial \Omega_{1}$ and $\partial \Omega_{2}$ was denoted ***t*_1_** and ***t*_2_** respectively, such that *t*_1_ + *t*_2_ = 0. Due to the assumed rigid nature of the anterior eye surface, it was taken as the master surface when defining contact. The Dirichlet boundary conditions were imposed by constraining the anterior eye in both displacement and rotation and by also preventing any X and Y displacement for the centre node of the lens. The extent to which the anterior eye was constrained was not considered to affect the accuracy of the results, as it has been demonstrated in clinical investigations that the eye shape had no short-term effect on soft contact lenses [19]. Through similar reasoning, it was deemed acceptable to model the cornea as two parallel surfaces, separated by a constant thickness of 545 µm as the eye was modelled as a rigid body [20].

The loading conditions were considered to include two uniform pressures, $P_{1}$ and $P_{2}$. The pressure $P_{1}$ was induced by the surface tension generated by the tears and was therefore only applied to the back surface of the contact lens, $\partial \Omega_{2,back},$ and the front surface of the eye, $\partial \Omega_{1,front}$. The value of $P_{1}$ was taken as 43.6 mPa [21]. $P_{2}$ was induced by the effect of eyelid blinking and took a value of 8.0 mmHg [22]. This pressure was applied incrementally to the front surface of the lens, $\partial \Omega_{2,front},$ halfway through the analysis step.

Due to the linearity of the stress–strain data produced in the tensile tests, the contact lenses were modelled as possessing both incompressible and linear elastic properties. This was achieved by assigning each material with a Poisson’s ratio of 0.49 and Young’s modulus corresponding to the value calculated from the experimental data. Eight-node trilinear hexahedral elements (HEX8) were utilised in both components of the model. The number of these elements used to model the eye and contact lens were 3280 and 2278, respectively. A mesh convergence study has demonstrated that this is the optimal number of elements to maximise accuracy whilst also not unnecessarily increasing computational time [23].

### 2.4. Contact Lens Design

The contact lens surfaces were designed with the use of a MATLAB program that calculated the element and nodal definitions necessary to produce a tri-curve lens with the desired optical power and geometric properties. The front and back surfaces of the lens were designed separately. In doing this, the front surface could be designed to the correct optical power and the back surface could be designed to ensure an optimal fit.

The custom-built lens design program allowed the user to input values of lens/peripheral zone diameter, D; lens base curve, $B_{c}$; spherical lens power (SPH); cylindrical lens power (CYL); refractive index, n; lens shape factor, ρ; central, T_c_, and edge, T_e_, thicknesses; and the diameter of the optic zone, d_1_. The range of values assigned to these variables are presented in Table 1. To gain a tri-curve lens design, the posterior lens surface was split into three zones, namely the optic zone (zone 1), the transient zone (zone 2), and the peripheral zone (zone 3). The radii of curvature, $R_{i,b},$ of each of the zones were computed, using the base curve, as:

(4) $$R_{1,b}=\;B_{c},\;R_{2,b}=B_{c}+2,\;\mathrm{and}\;R_{3,b}=B_{c}-2.$$

The centres of each of the radii of curvature were computed by taking the x components, $X_{c,i},$ as zero and the z components, $Z_{c,i},$ as:

(5) $$Z_{c,1}=-R_{1,b},$$

(6) $$Z_{c,2}=Z_{c,1}-R_{2,b}\cos\left(\sin^{-1}\left(\frac{d_{1}}{2R_{2,b}}\right)\right)+R_{1,b}\cos\left(\sin^{-1}\left(\frac{d_{1}}{2R_{1,b}}\right)\right),$$

(7) $$Z_{c,3}=Z_{c,2}-R_{3,b}\cos\left(\sin^{-1}\left(\frac{d_{2}}{2R_{3,b}}\right)\right)+R_{2,b}\cos\left(\sin^{-1}\left(\frac{d_{2}}{2R_{2,b}}\right)\right),$$

**Figure 4:**
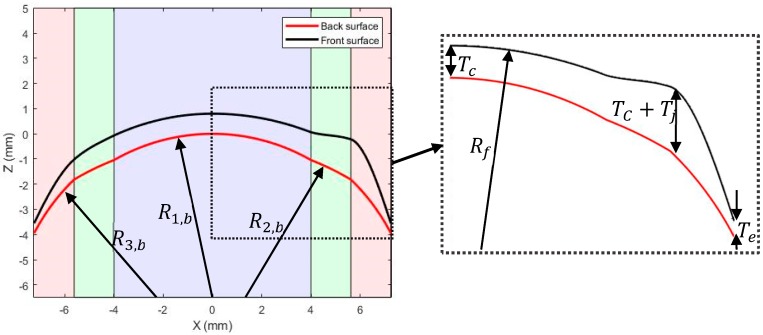
Geometric parameters utilised in the contact lens design process. The optic, transient, and peripheral zones are represented by the purple, green, and pink shaded areas, respectively.

These values were then used to inform the calculation of the back-surface height, $Z_{b}$:

(8) $$Z_{b}=\{\begin{array}{c} Z_{c,1}+\sqrt{R_{1,b}^{2}-X^{2}}\;within\;the\;optic\;zone\\ Z_{c,2}+\sqrt{R_{2,b}^{2}-X^{2}}\;within\;the\;transient\;zone\\ Z_{c,3}+\sqrt{R_{3,b}^{2}-X^{2}}\;within\;the\;peripheral\;zone \end{array},$$

where X refers to the horizontal distance from the apex, Figure 4. The back surface of the lens was rotational symmetric. This allowed the two-dimensional surface to be rotated through 180° to achieve the complete three-dimensional topography.

Following the initial design of the lens posterior, the front surface was designed to achieve the required refractive power. The maximum and minimum lens powers were computed as:

(9) $$P_{max}=\max\left(SPH+CYL,\;SPH\right),$$

(10) $$P_{min}=\min\left(SPH+CYL,\;SPH\right),$$

A one-dimensional set, $P_{0},$ containing the lens powers across 360 equally spaced meridians was then deduced by using a cosine wave with domain [0, 4π] to vary between the maximum and minimum values of power:

(11) $$P_{0}=P_{amp}\cos\left(\theta \right)+P_{mean},$$

where $P_{mean}$ and $P_{amp}$ are mean and the difference between the mean and the maximum power respectively, and θ is a set containing 360 equally spaced values from 0 to 4π. The values $P_{0}$ were then updated by using piecewise cubic interpolation to incorporate the axis of cylinder into the meridian angles so that the maximum values of lens power occur in the correct locations. Following this, the radius of curvature of the front surface, $R_{f},$ was computed for each of the 360 meridians using the Lens Maker’s Equation [24]:

(12) $$R_{j,f}=\frac{T_{c}{\left(n-1\right)}^{2}+n\left(n-1\right)R_{1,b}}{nR_{1,b}P_{j,0}+n\left(n-1\right)},$$

where j refers to the jth meridian. A set containing the front surface elevations across a single meridian was then computed as:

(13) $$Z_{j,f}=T_{c}-\frac{1}{\rho }\left(R_{j,f}-\sqrt{R_{j,f}^{2}-\rho X^{2}}\right),$$

**Figure 5:**
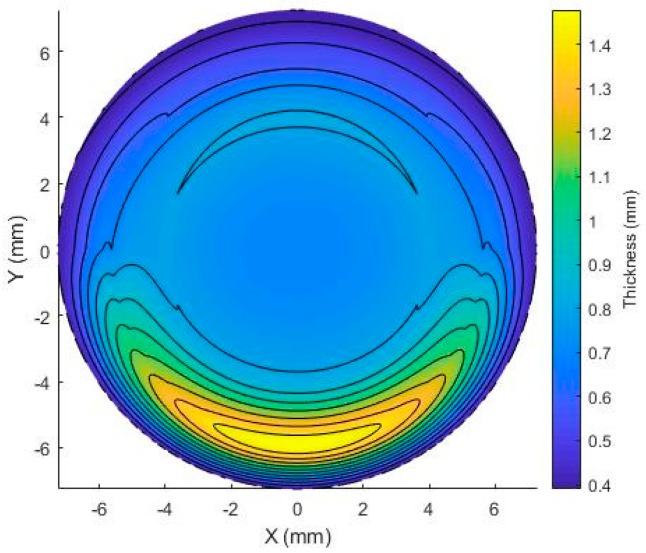
Graphical representation of the variation in lens thickness due to the inclusion of a single ballast.

Due to the lack of rotational symmetry in the front surface, this step was repeated for each meridian. The design of the front surface was finalised by adding a single ballast to the lower portion of the lens, Figure 5. This was achieved by selectively increasing the thickness of the lens. The following relation was utilised for the increase in lens thickness:

(14) $$T_{j}=T_{c}(1-W\sin\theta ),$$

where $T_{j}$ is the added thickness, θ is the meridian angle, and W is a weighting factor that allows for the selective placement of the ballast:

(15) $$W=\{\begin{array}{l} 0.2,\;0\leq \theta \leq \pi \\ 1.0,\;\pi <\theta <2\pi \end{array},$$

For certain values of spherical and cylindrical lens powers, it became apparent that the curvature required in the front surface was causing the two surfaces to intersect each other, leading to negative element volumes when they were later modelled in FEBio finite element software (The University of Utah, Salt Lake City, USA & Columbia University, New York, NY, USA). This problem was overcome by adding 0.01 mm to the central thickness, updating the elevation of the front surface, and checking that the thickness in all areas of the lens was greater than a minimum value of 0.1 mm. This process was repeated until the minimum thickness had been exceeded. Although this altered the overall thickness of the lenses, it was not considered to affect the refractive properties as the curvature of the front surface remained unchanged throughout.

Each contact lens design was fitted to the same corneal model. The curvature of this corneal model was 43.7 D. This corresponds to the average curvature value observed in the study conducted by Gilani et al. [15].

### 2.5. Light Ray Tracing

Assessment of the EPC of the fitted soft contact lens was conducted using a three-dimensional ray tracing code, built in the MATLAB software package (MathWorks, Natick, MA, USA). The light ray tracing process began by exporting the modified coordinates of the soft contact lens from the FEBio program. These coordinates were then fitted to a set of two surfaces, f, corresponding to the lens interfaces. The refractive indices of the lenses were then defined in accordance with the values provided by Contamac (Saffron Walden, England, UK), Table 2.

Same levelled light sources were introduced above the front surface of the contact lens, with positions $S_{i}=\left(x_{i},y_{i},z_{i}\right)$, where i=1, 2, 3, …., n, for a total of *n* light sources. A set of incoming light rays each with initial positions $S_{i}$ and normalised direction vector $d_{i}=\left(d_{xi},d_{yi},d_{zi}\right)$ were introduced such that they were travelling parallel to the optical axis of the contact lens $\left(d_{xi}=d_{yi}=0\right)$. At the point at which each light ray intersected the surface of the lens, the angle of incidence was determined by calculating the angle between the normal vector, $N_{i},$ to the surface and the angle of the incoming ray. Due to the implicit definition of the surface, the normal vector could be determined by evaluating the following relation at the point at which refraction was occurring [25]:

(16) $$N_{i}=\;\frac{\nabla f}{\left\|\nabla f\right\|},$$

Following this, the angle of incidence, $\phi_{air}$, for each ray was determined by computing the dot product between the normal and directional vectors:

(17) $$\phi_{air_{1},i}=\cos^{-1}\left(\frac{d_{i}\cdot N_{i}}{\left\|d_{i}\|\|N_{i}\right\|}\right),$$

**Figure 6:**
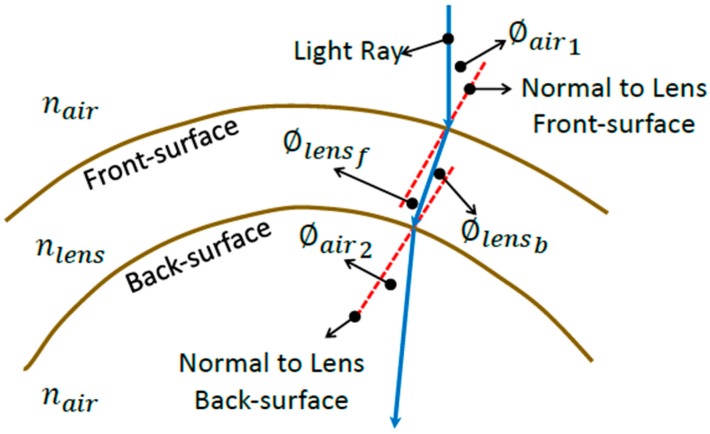
Diagram depicting the refraction process through the soft contact lens in a two-dimensional sketch for simplification purposes.

Snell’s law was then utilised to compute the angle at which the refracted ray travelled, as it passed through the depth of the lens [26]. A two-dimensional depiction of this is shown in Figure 6:

(18) $$\phi_{lens_{f},i}=\sin^{-1}\left(\frac{n_{air}}{n_{lens}}\sin\left(\phi_{air,i}\right)\right),$$

The coordinate axis was rotated such that the y component of the unit normal vector, ${\hat{N}}_{i},$ was zero. This was done by computing the meridian angle, $\theta_{mer},$ at which refraction occurred from the x (${\hat{N}}_{x,i})$ and y ${(\hat{N}}_{y,i})$ components of the unit normal vector:

(19) $$\theta_{mer}=\tan^{-1}\left(\frac{{\hat{N}}_{y,i}}{{\hat{N}}_{x,i}}\right),$$

This angle was then used, with the rotation matrix for rotation about the z-axis, to compute the x and z components of the normal vector in the new coordinate system.

(20) $${\hat{N}}_{new,i}^{\prime }=\left(\begin{array}{ccc} \cos\theta_{mer} & \sin\theta_{mer} & 0\\ -\sin\theta_{mer} & \cos\theta_{mer} & 0\\ 0 & 0 & 1 \end{array}\right){\hat{N}}_{i}^{\prime },$$

The new unit normal vector, ${\hat{N}}_{new,i},$ was then rotated about the origin by $\frac{\pi }{2}$, converted into polar coordinates, rotated by the angle of refraction, and then converted back into Cartesian coordinates to yield the updated unit direction vector, with respect to the new coordinate system. The updated unit direction vector was then rotated about the z-axis, in order to obtain the unit direction vector with respect to the original coordinate system. This process was repeated for each of the incident light rays as they encountered the refraction interfaces.

Following the refraction of each of the rays through the two lens interfaces, the focal point of each ray was determined by locating the position at which the refracted light ray intersected the optical axis. The distance between the average point of intersection and the lens apex was then calculated as the focal length, f, which was then inverted to deduce the effective power change (EPC) due to the presence of the lens [27].

## 3. Statistical Analysis

Statistical analysis was conducted on the results presented in this paper through the use of the MATLAB Statistics and Machine Learning Toolbox. The p-values were calculated, for pairs of data sets, using a two-sample t-test with a significance level of 5%. This choice of significance level allows the observed effects to be characterised as significant if they are less than or equal to 0.05. In addition to the significance, the correlation coefficients were also computed for each of the materials with respect to the changes in lens power. Due to the difference in behaviour between the observed EPC data, statistical analysis was carried out separately for each of the defined regions.

### 3.1. Results

#### 3.1.1. Material Properties

**Figure 7:**
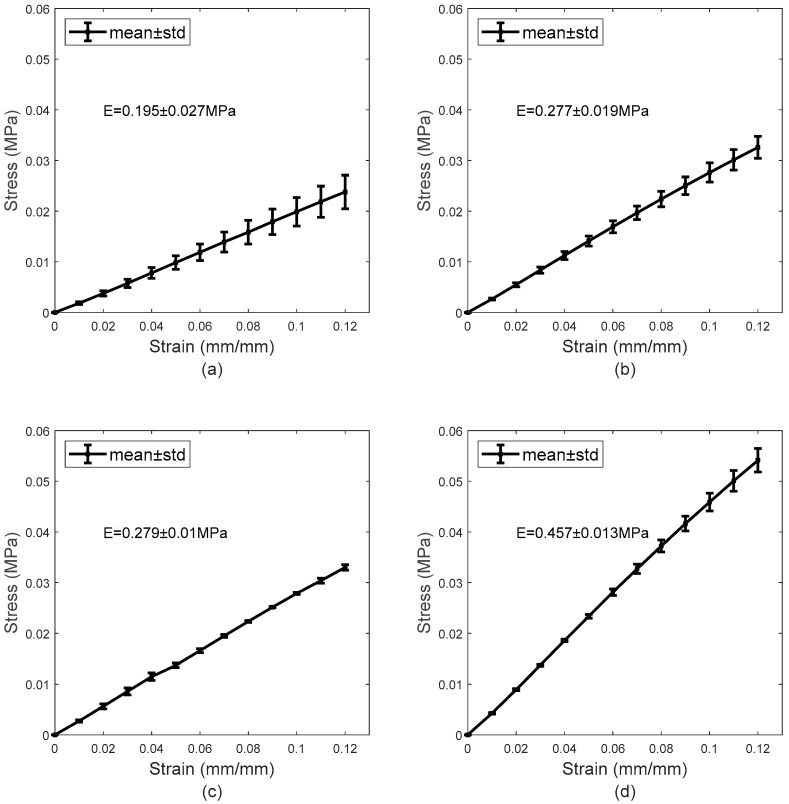
Mean stress–strain curves obtained during the tensile test for H77p0 (**a**), SiH74p5-blue (**b**), SiH74p5-clear (**c**), and H64p0 (**d**).

The data obtained in the uniaxial tensile testing procedure are shown in Figure 7. By calculating the gradient of the trend line produced from the data points, the moduli of elasticity were calculated as 0.195 ± 0.027 MPa, 0.277 ± 0.019 MPa, 0.279 ± 0.01 MPa, and 0.457 ± 0.013 MPa for the H77p0, SiH74p5-blue, SiH74p5-clear, and H65p0 materials, respectively. The Young’s moduli of the latter three differ considerably from those provided by Contamac, Table 2. Despite this, after considering the number of repeat readings and the fact that each of the coefficients of variation is far less than one, this was not deemed to be problematic. An increase in water content is consistent with a significant reduction in Young’s modulus, Table 3.

#### 3.1.2. Spherical Lens Results

**Figure 8:**
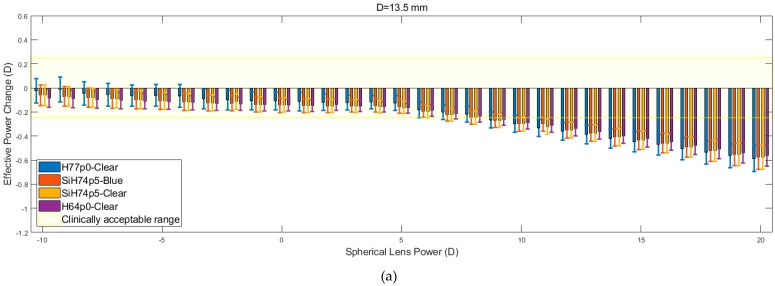

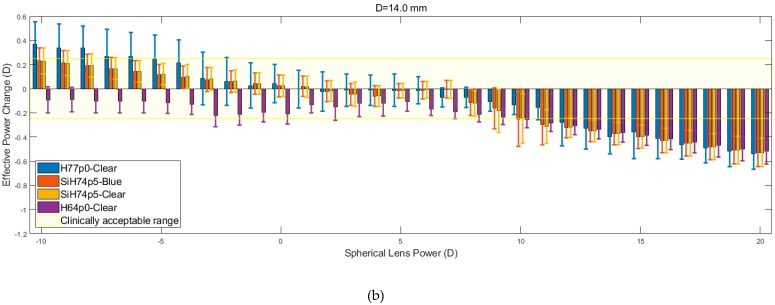

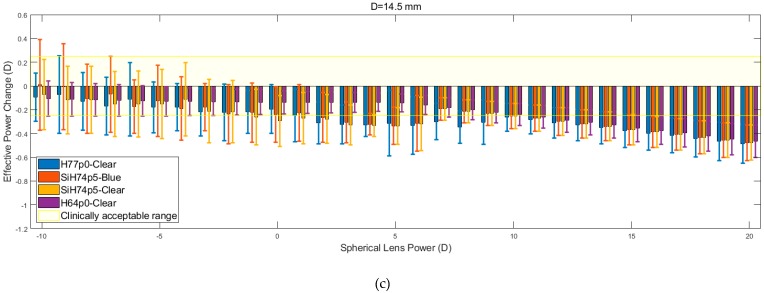
Effective power changes in spherical lenses for each of the four hydrogel materials. Results are presented for lens diameters D of 13.5 mm (**a**), 14.0 mm (**b**), and 14.5 mm (**c**).

The results obtained in the analysis of the spherical lens fitting are shown in Figure 8. For each of the corneal diameters, it is observed that the recorded values of the EPC decrease as the spherical lens power increases. This reduction in EPC is less apparent for lower spherical lens power, specifically those in the range of −10 to 5 D. Within this range, the rate at which EPC declines is halted by constant fluctuations. As the nominal lens power is increased beyond this initial range, the fluctuations are far less apparent, and the EPC tends to decline linearly at an increased rate.

The results show that the H64p0-clear material was far more resilient to changes in EPC across the initial range (−10 to +5 D). However, lenses composed of this material, along with those made up of the two SiH74p5 silicone hydrogels, consistently left the clinically acceptable range at lower spherical lens powers compared to the H77p0-clear hydrogel lenses. Following the reduction of EPC beyond the acceptable range, the four materials demonstrated almost identical behaviour (*p* > 0.05 in all but one comparison, Table 4), whereby they each yielded a linear decrease in EPC, with roughly equal gradients. It was also observed that the EPC for each material tended to converge to a value of −0.5 D at a lens power of +20 D.

#### 3.1.3. Cylindrical Lens Results

**Figure 9:**
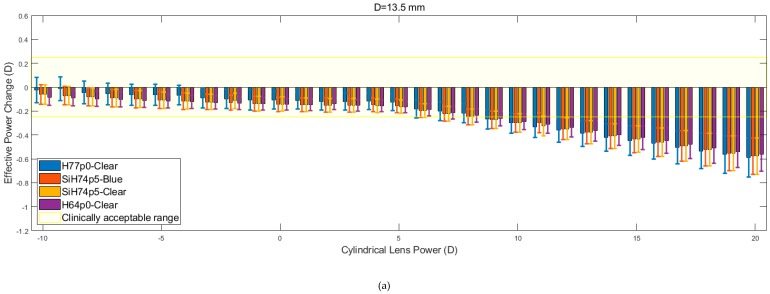

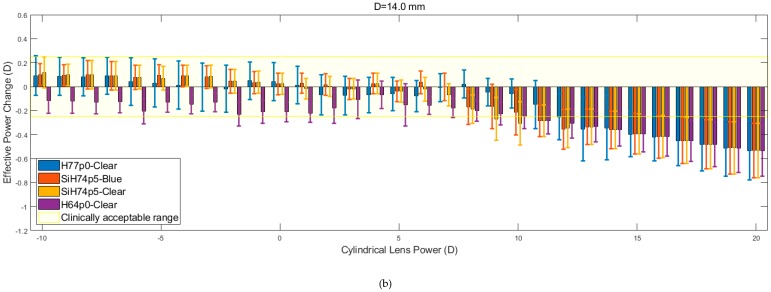

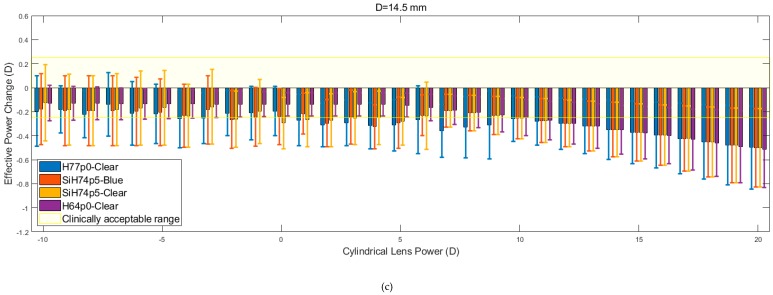
Effective power changes in cylindrical lenses for each of the four hydrogel materials. Results are presented for lens diameters D of 13.5 mm (**a**), 14.0 mm (**b**), and 14.5 mm (**c**).

Figure 9 shows the relation between EPC and cylindrical lens power for three different corneal diameters. Despite the apparent similarity, it is evident that within the initial range of cylindrical lens powers (−10 to + 5 D), the rate at which EPC declines is consistently lower than the rate that is observed from the spherical lens data. When this is coupled with the observed reduction in the magnitude of the fluctuations, the consistency of the results increases. As was observed for the spherical lenses, the H77p0-clear hydrogel generally remained within the clinically acceptable EPC range, for a larger range of lens powers, when compared to the other hydrogel lenses. Additionally, as was observed with the spherical lenses, the recorded values of EPC for each material, tended to converge and decrease far more linearly as the lens power was increased beyond 5 D. 

## 4. Discussion

In this paper, the magnitude of the effective power change for soft contact lenses, with varying lens power, was determined for lenses composed of four different hydrogel materials. This investigation was conducted by determining the material properties of each hydrogel material through tensile testing, modelling the contact lenses fitting process using finite element analysis, and using light raytracing analysis, to measure the effective power change induced by the lens. A clinically acceptable EPC was included in the analysis as |ΔP|≤0.25 [28,29]. This value was determined by considering the fact that, in clinical practice, trial lenses are varied in increments of 0.25 D.

The material properties obtained from the tensile test differ to the empirical values provided by a manufacturer of contact lenses material, Contamac (Saffron Walden, England, UK). Although this may be due to experimental error, the most likely cause of this difference is the fact that, in this study, the samples were only subjected to tensile loads which would be expected to occur in vivo. When compared to the procedure employed by Contamac, whereby the materials were tested until failure, the difference in material performance was deemed to be acceptable. The data obtained demonstrate that the hydrogel materials are linear elastic in the range of loading conditions expected in vivo and that a higher Young’s modulus is consistent with higher water content. This was highlighted by the significantly higher Young’s modulus produced by the H64p0 data, when compared to all other tested materials, Table 3. These findings are in accordance with previous studies [5,30].

The values of EPC produced by each of the four hydrogel materials tended to converge to the same value and decrease linearly as lens power was increased towards the higher end of the lens power spectrum (5–20 D). This is demonstrated by the consistently high p-values that were calculated for this region, Table 4. This suggests that for higher lens powers (>5 D), the material choice may not be a significant factor in the overall effective power change. For lens powers below this range, however, strikingly different behaviour is observed. The EPC data present in this region (−10 D < lens power < 5 D) is characterised by repeated fluctuations.

These fluctuations are far less prominent in the H64p0 material, which remains at a roughly constant EPC value of −0.1 across the entire lower range. This suggests that materials with higher moduli of elasticity may be more resilient to changes in EPC when considering negative and relatively low values of lens powers. This is in accordance with the findings in Hall et al. [31] that demonstrated through experimental methods that the effective power change induced by stiffer hydrogel lenses was far easier to predict than the EPC in those with relatively lower stiffness.

Additionally, when compared to the higher end of the lens power spectrum, a significantly lower rate of change in EPC with an increase in lens power is observed. This is particularly prominent in the data obtained from the cylindrical lenses (average correlation factor of −0.812 in the lower range versus −0.965 in the higher, Table 5). This increase in the magnitude of EPC for higher lens powers is due to the fact that, in general, lenses of higher power tend to be more reliant on excessive concavity and higher thicknesses and, upon conforming to the shape of the eye, experience a larger percentage change in their dimensions. This indicates that as lens power enters the higher end of the spectrum, EPC has an increasing and decreasing reliance on geometric and material properties, respectively. 

The limitations associated with this study are the use of only three lens diameters and the fact that corneal geometry was kept constant throughout. The effect of corneal geometry on EPC has been demonstrated previously by Abass et al. [23]. Upon consideration of their data, it was concluded that varying corneal geometry would display the same trends, except with a slight positive or negative shift in the EPC data. As the aim of this study was to deduce the effect of varying the material properties, the exclusion of this factor was deemed to be acceptable. In the future, this study will be extended to different types of clinically available soft contact lenses and include models of the cornea with dimensions that are obtained directly from clinical data. In doing this, the understanding of how lenses should be prescribed on a patient-specific basis will be improved, thus enhancing patient health and satisfaction.

## Figures and Tables

**Table 1:** Geometrical properties used to inform the contact lens design process.

Lens Diameter, D (mm)	13.5, 14.0, 14.5
Optic Zone Diameter, $d_{1}$ (mm)	8.0
Base Curve, $B_{c}$ (mm)	8.5
Spherical Lens Power (D)	−20.0 to 10.0 (increments of 1.0)
Cylindrical Lens Power (D) @ 90°	−20.0 to 10.0 (increments of 1.0)
Lens Shape Factor, ρ	0.75
Central Thickness, $T_{c}$ (mm)	0.25
Edge Thickness, $T_{e}$ (mm)	0.4

**Table 2:** Empirical data provided by Contamac for each of the four hydrogel materials.

Material Short Name	H77p0-Clear	SiH74p5-Blue	SiH74p5-Clear	H64p0-Clear
Base material	Hydrogel	Silicone Hydrogel	Silicone Hydrogel	Hydrogel
Material full commercial name	Contaflex Clear UV 77–Filcon II 3	Definitive (V3) 74% Blue UV (SiH)–Filcon V3-Efrofilcon A	Definitive (V3) 74% Clear (SiH)–Filcon V3-Efrofilcon A	Contaflex Clear UV 67–Filcon II 2–Zylofilcon A
Water Content	77%	74.5%	74.5%	64%
Refractive Index (wet)	1.3739	1.3753	1.3749	1.3920
Modulus of Elasticity (MPa)	0.17	0.35	0.35	0.37

**Table 3:** Significance p-values for comparison between the experimentally measured Young’s moduli of each of the four tested materials. The results used to generate these values are presented in Figure 7.

	H77p0-Clear	SiH74p5-Blue	SiH74p5-Clear	H64p0-Clear
**H77p0-Clear**	-	*p* < 0.001	*p* < 0.001	*p* < 0.001
**SiH74p5-Blue**	*p* < 0.001	-	*p* = 0.449	*p* < 0.001
**SiH74p5-Clear**	*p* < 0.001	*p* = 0.449	-	*p* < 0.001
**H64p0-Clear**	*p* < 0.001	*p* < 0.001	*p* < 0.001	-

**Table 4:** Significance values for comparing the effective power change (EPC) results for each of the four materials. The EPC in each lens power region was recorded for each lens material, type (spherical or cylindrical), and diameter. These values were then used to compute the significance provided within the table. Due to the difference in behaviour in the two regions of each graph, the significance values of these regions were calculated separately. Results used for comparison are presented in Figure 8 and Figure 9.

								Key:	*S*	Spherical
									*C*	Cylindrical
	Lens Power < 5 D						Lens Power ≥ 5 D			
D = 13.5 mm	H77p0-Clear	SiH74p5-Blue	SiH74p5-Clear	H64p0-Clear		D = 13.5 mm	H77p0-Clear	SiH74p5-Blue	SiH74p5-Clear	H64p0-Clear
H77p0-Clear	-	*C p* < 0.001	*C p* < 0.001	*C p* < 0.001		H77p0-Clear	-	*C p* = 0.997	*C p* = 0.997	*C p* = 0.942
SiH74p5-Blue	*S p* = 0.006	-	*C p* = 0.764	*C p* = 0.246		SiH74p5-Blue	*S p* = 0.992	-	*C p* = 0.994	*C p* = 0.943
SiH74p5-Clear	*S p* = 0.006	*S p* = 0.998	-	*C p* = 0.375		SiH74p5-Clear	*S p* = 0.965	*S p* = 0.955	-	*C p* = 0.937
H64p0-Clear	*S p* < 0.001	*S p* = 0.464	*S p* = 0.474	-		H64p0-Clear	*S p* = 0.877	*S p* = 0.879	*S p* = 0.833	-
D = 14.0 mm	H77p0-Clear	SiH74p5-Blue	SiH74p5-Clear	H64p0-Clear		D = 14.0 mm	H77p0-Clear	SiH74p5-Blue	SiH74p5-Clear	H64p0-Clear
H77p0-Clear	-	*C p* = 0.043	*C p* = 0.075	*C p* < 0.001		H77p0-Clear	-	*C p* = 0.701	*C p* = 0.487	*C p* = 0.278
SiH74p5-Blue	*S p* = 0.169	-	*C p* = 0.840	*C p* < 0.001		SiH74p5-Blue	*S p* = 0.721	-	*C p* = 0.761	*C p* = 0.502
SiH74p5-Clear	*S p* = 0.180	*S p* = 0.956	-	*C p* <0.001		SiH74p5-Clear	*S p* = 0.700	*S p* = 0.977	-	*C p* = 0.721
H64p0-Clear	*S p* < 0.001	*S p* < 0.001	*S p* < 0.001	-		H64p0-Clear	*S p* = 0.339	*S p* = 0.566	*S p* = 0.588	-
D = 14.5 mm	H77p0-Clear	SiH74p5-Blue	SiH74p5-Clear	H64p0-Clear		D = 14.5 mm	H77p0-Clear	SiH74p5-Blue	SiH74p5-Clear	H64p0-Clear
H77p0-Clear	-	*C p* = 0.750	*C p* = 0.320	*C p* < 0.001		H77p0-Clear	-	*C p* = 0.396	*C p* = 0.319	*C p* = 0.190
SiH74p5-Blue	*S p* = 0.498	-	*C p* = 0.450	*C p* < 0.001		SiH74p5-Blue	*S p* = 0.334	-	*C p* = 0.886	*C p* = 0.577
SiH74p5-Clear	*S p* = 0.880	*S p* = 0.429	-	*C p* < 0.001		SiH74p5-Clear	*S p* = 0.351	*S p* = 0.976	-	*C p* = 0.668
H64p0-Clear	*S p* = 0.002	*S p* = 0.075	*S p* = 0.002	-		H64p0-Clear	*S p* = 0.039	*S p* = 0.228	*S p* = 0.218	-

**Table 5:** Correlation factors between the EPC values produced by each material and the associated lens powers.

	Lens Power < 5 D						Lens Power ≥ 5 D			
D = 13.5 mm	H77p0-Clear	SiH74p5-Blue	SiH74p5-Clear	H64p0-Clear		D = 13.5 mm	H77p0-Clear	SiH74p5-Blue	SiH74p5-Clear	H64p0-Clear
Spherical	−0.9634	−0.9698	−0.9816	−0.9812		Spherical	−0.9985	−0.998	−0.9995	−0.9992
Cylindrical	−0.9115	−0.9004	−0.9594	−0.9973		Cylindrical	−0.9972	−0.9986	−0.9987	−0.9985
D = 14.0 mm	H77p0-Clear	SiH74p5-Blue	SiH74p5-Clear	H64p0-Clear		D = 14.0 mm	H77p0-Clear	SiH74p5-Blue	SiH74p5-Clear	H64p0-Clear
Spherical	−0.9674	−0.9973	−0.9987	−0.4511		Spherical	−0.9858	−0.9773	−0.975	−0.9982
Cylindrical	−0.8687	−0.9084	−0.9099	−0.1359		Cylindrical	−0.9526	−0.9696	−0.9657	−0.9973
D = 14.5 mm	H77p0-Clear	SiH74p5-Blue	SiH74p5-Clear	H64p0-Clear		D = 14.5 mm	H77p0-Clear	SiH74p5-Blue	SiH74p5-Clear	H64p0-Clear
Spherical	−0.9461	−0.9519	−0.9559	−0.9142		Spherical	−0.8342	−0.8286	−0.8227	−0.9993
Cylindrical	−0.7581	−0.8186	−0.7896	−0.7896		Cylindrical	−0.828	−0.9318	−0.9399	−0.9978
